# Prolapsed sigmoid intussusception per anus in an elderly man: a case report

**DOI:** 10.1186/1752-1947-5-389

**Published:** 2011-08-17

**Authors:** Penn S Teyha, Alphonce Chandika, Vihar R Kotecha

**Affiliations:** 1 Department of Surgery, Bugando Medical Centre, P.O.Box 1370, Mwanza, Tanzania; 2 Department of Surgery, Weill Bugando University College of Health Sciences, P.O.Box 1464, Mwanza, Tanzania

## Abstract

**Background:**

Intussusception in pediatrics is widely documented and well described. On the basis of the literature, however, adult intussusception is a rare entity with a prevalence of from 1% to 5%. The majority of adult patients with intussusception have an underlying pathology that needs to be identified by performing a proper physical examination and a wide array of investigations.

**Case presentation:**

We present a case of a 66-year-old African man who presented to our emergency department with a mass protruding per anus with obstipation. During laparotomy, we found that the sigmoid colon had intussuscepted into the rectum and out from the anus. Other abdominal viscera were normal and without any obvious mesenteric lymphadenopathy. Sigmoid colectomy and spectacle colostomy were performed. Grossly, the excised bowel looked normal, but the histologic results showed features of necrosis and chronic inflammation.

**Conclusion:**

While 70% to 90% of cases of adult intussusception have an identifiable cause or lesion, most pediatric intussusceptions are idiopathic. The presentation in an adult described herein was of an uncommon idiopathic type with no identifiable cause found on the basis of the history, physical examination, or histological findings.

## Background

"Intussusception" refers to the telescoping of the proximal bowel into the distal bowel or *vice versa *(retrograde intussusception). The telescoped part is referred to as the "intussusceptum," and the receiving part is called the "intussuscipiens." This condition is commonly described in infants and rarely in adults. Adult intussusception represents 5% of all cases of intussusception and accounts for only 1% to 5% of intestinal obstructions in adults [1,2]. Almost 90% of cases of intussusception in adults have an underlying bowel pathology such as carcinoma, polyps, Meckel's diverticulum, colonic diverticulum, strictures, or benign neoplasms, which are usually discovered intra-operatively [3]. In this report, we present a case of an elderly man with a prolapsed intussusception.

## Case presentation

A 66-year-old African man from a rural area presented to our emergency department complaining of persistent, dull lower-back pain of three months' duration and a mass protruding per anus for the previous four days. His lower-back pain was radiating to both thighs and was aggravated by farming activities. His pain was relieved by resting, and the pain was not accompanied by numbness of the limbs. He had no history of difficulty in micturition. The mass protruded per anus spontaneously while he defecated and was associated with severe pain followed by obstipation. There was no obvious bleeding per rectum upon his presentation to our emergency department. He had no history of constipation prior to the protrusion of the mass. He had no history of on-and-off mass protrusion per anus even when lifting loads. In addition, he had no history of changes in bowel habits or blood-stained stools, no history of tenesmus, and no history of incomplete bowel emptying post-defecation. He had no history of feeling any abdominal swelling and no history of significant weight loss. An attempt to reduce the mass was made at the district hospital, but the procedure failed; hence the patient was referred to Bugando Medical Centre. His medical history was uneventful.

**Figure 1:**
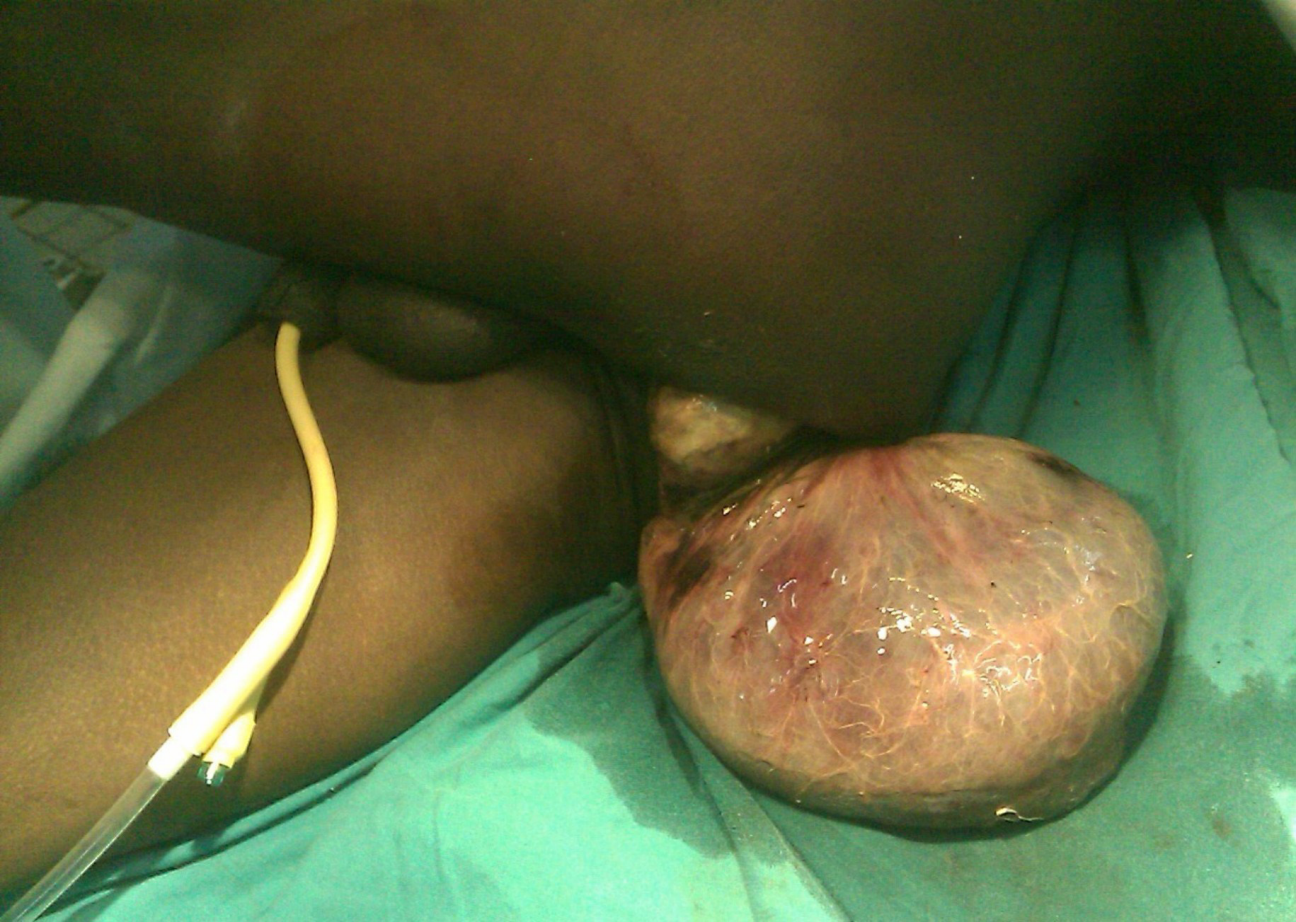
**The prolapsed bowel portion from the anus**.

**Figure 2:**
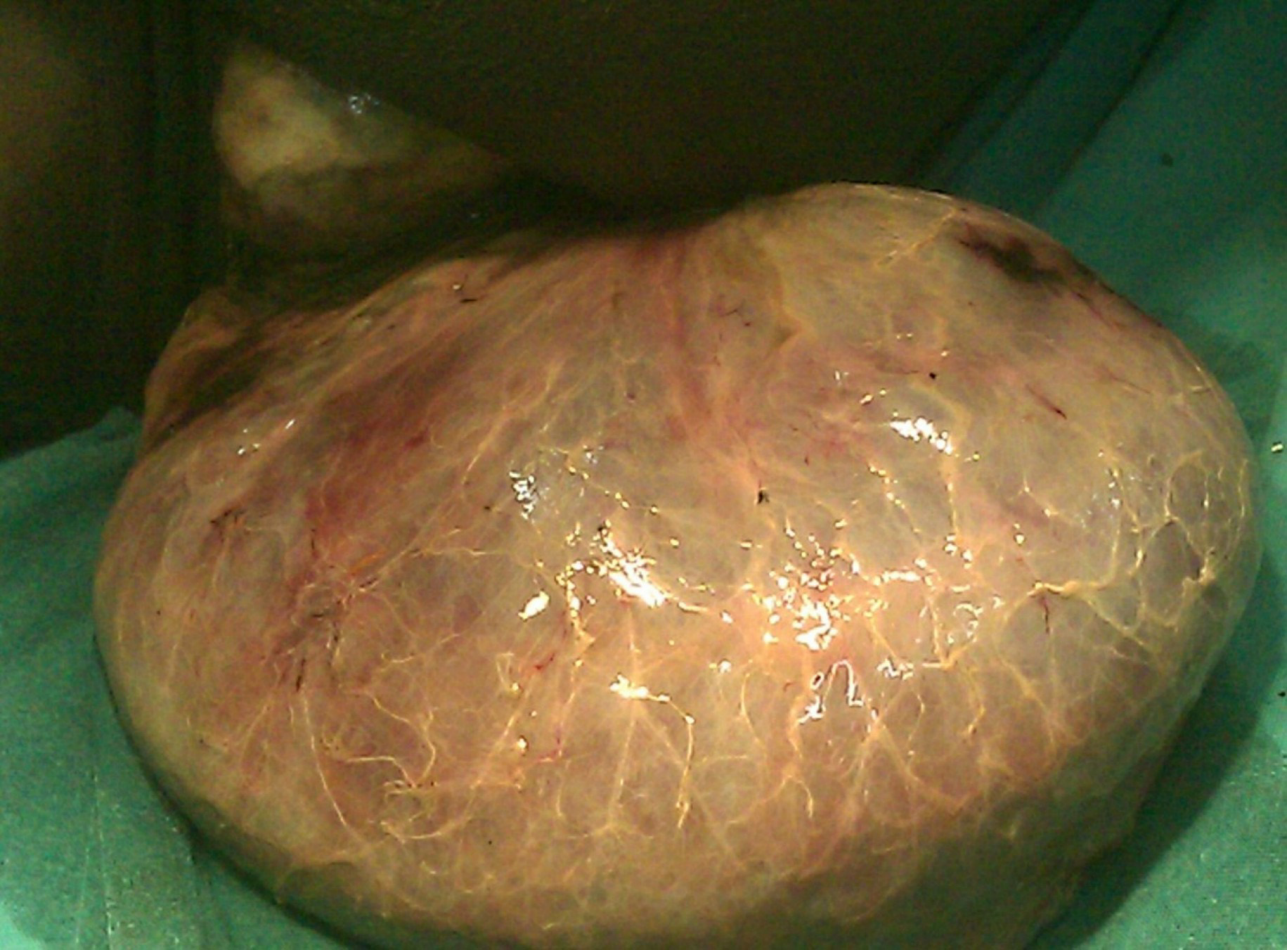
**Closer view of the prolapsed bowel with foci of hemorrhage**.

His physical examination revealed that he was in pain, alert, afebrile (body temperature 37°C), and dehydrated, but he was not pale. His blood pressure was stable at 100/70 mmHg, and his radial pulse was 90 beats/minute, weak, regular, and synchronous with other pulses. He had no cervical lymphadenopathy, and no Virchow's node was palpable. He had a scaphoid abdomen that moved with respiration, but no visible peristalsis was observed, his abdomen was not tender, and no obvious mass was palpable. His liver, spleen, and kidneys were not palpable, and a tympanic percussion note was heard throughout the abdomen. His bowel sounds were increased on auscultation. The digital rectal examination revealed a large, foul-smelling, gangrenous sigmoid-shaped mass protruding per anus (Figures 1 and 2). The rest of his systemic examination was non-contributory.

**Figure 3:**
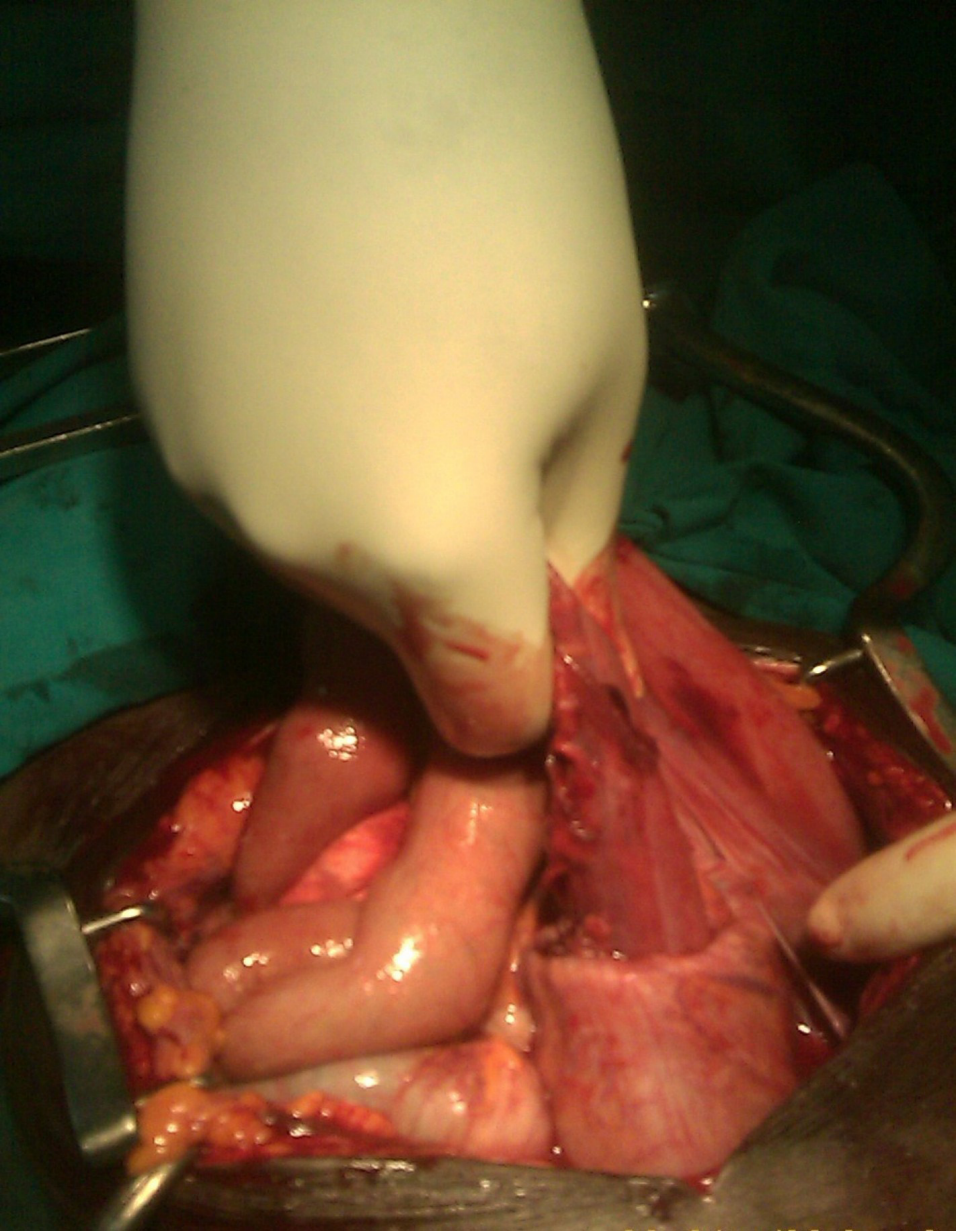
**Finger indicating the intussusception site**.

**Figure 4:**
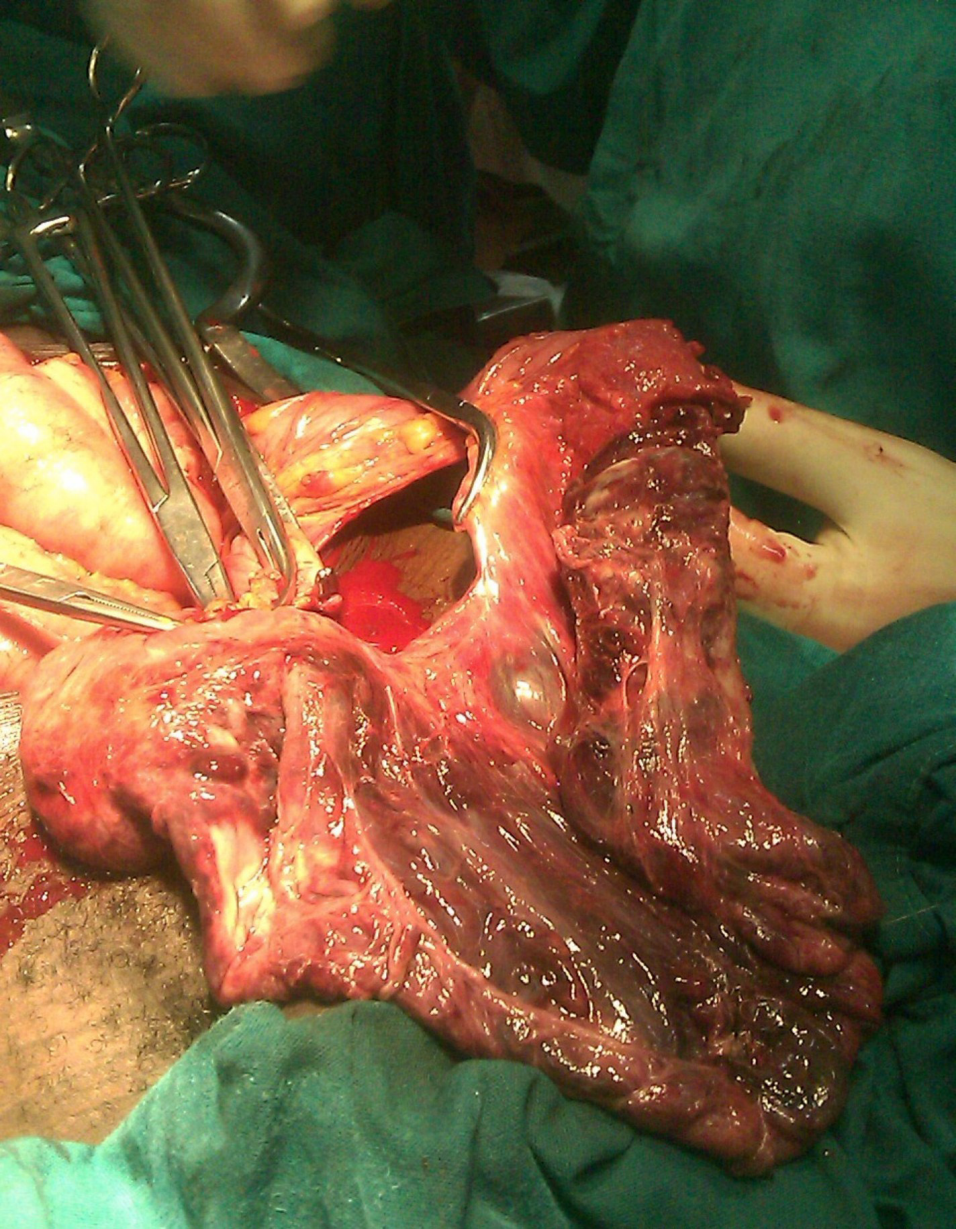
**The gangrenous sigmoid colon that was removed during surgery**.

A provisional diagnosis of sigmoid intussusception with a differential diagnosis of rectal prolapse was made. Blood samples were taken to measure his hemoglobin level and blood grouping. His hemoglobin level was 10 g/dL. Pre-operatively the patient received 3 L of Ringer's lactate solution over the course of one hour, intravenous ceftriaxone 1 g, and metronidazole 500 mg, and he was prepared for emergency exploratory laparotomy. The abdomen was approached through an extended mid-line incision. The intra-operative findings were that the sigmoid colon had telescoped into the rectum and out per anus (Figure 3). The large bowel was not dilated. No other mass or pathology was identified intra-abdominally, nor were mesenteric lymph nodes palpable. Milking of the intussusceptum was done followed by resection of the gangrenous sigmoid colon (Figure 4), and the two bowel ends were exteriorized to form a spectacle colostomy. Macroscopically, normal rugae with no visible lesions or hemorrhagic serosa were visualized. The microscopic findings revealed features of chronic inflammation and necrosis.

## Discussion

Intussusception in adults is an uncommon condition, representing 1% of cases of adult bowel obstruction and less than 1% of hospital admissions [4]. It is a common cause of bowel obstruction in infants, in whom it presents with a classic triad of symptoms and signs: crampy abdominal pain, a palpable sausage-shaped mass mainly in the right upper quadrant, and currant jelly stools [5]. In one case series, it was noted that adult intussusception was slightly more predominant among men, with a male:female ratio of 1.8:1 [1]. Adult intussusception may present with acute, sub-acute, or chronic non-specific features, which makes its diagnosis difficult. In our patient, sub-acute non-specific complaints of backache during strenuous activity culminated in acute prolapsed intussusception of the sigmoid. In one series, computed tomography, with an accuracy of 58% to 100%, was the most efficient tool in diagnosing intussusception, followed by abdominal ultrasound [1,2]. In our patient, the mass was obviously protruding from the anus and did not warrant any complex investigations. Our case could possibly have been confused with complete rectal prolapse. A retrospective study by Rashid and Basson [6] showed that patients with rectal prolapse exhibited a 4.2-fold relative risk for colorectal cancer compared with the comparative group. In our patient, the diagnosis of colorectal cancer was at the top of the list as the underlying cause of intussusception, mainly because of his age at presentation. With regard to the management of adult sigmoid intussusception, several schools of thought exist. However, there is a common consensus that the treatment of choice is resection of the affected portion of the sigmoid colon, as the results reported in several series have revealed that 90% of cases have an underlying pathology. Lynn and Agrez [7] reported the case of a patient with sigmoid colon intussusception in whom the rectum was opened circumferentially by using diathermy at the point of the intussusception and the intussuscepted sigmoid colon was removed from the rectum through the anus. However, this procedure could cause contamination of the abdominal cavity. In our case, given that the intussusceptum was edematous and gangrenous, a longitudinal incision was made on the prolapsed bowel to facilitate reduction with a milking motion. A decision to perform reduction was made after assessing the abdominal viscera and the presence of mesenteric lymph nodes for any macroscopic evidence of a large bowel tumor. We did a spectacle colostomy after resection of the gangrenous bowel, as the viability of the sigmoid colon could not be guaranteed for primary end-to-end anastomosis.

While 70% to 90% of adult intussusceptions have an identifiable cause or lesion, most pediatric intussusceptions are idiopathic [1,2]. The case of an elderly patient presented here was of the uncommon idiopathic type with no identifiable cause found in the history, physical examination, or histological findings.

## Conclusion

Intussusception is a well-described condition that has been documented mostly in pediatric patients. Adult intussusception is a rare entity. Prolapsed intussusception per anus has rectal prolapse as its most likely differential diagnosis; hence sigmoid prolapse has to be kept in mind, since adult intussusception usually has an underlying cause. All stigmata for the cause should be ruled out on the basis of the patient's history and physical examination as well as during laparotomy.

## Consent

Written informed consent was obtained from the patient for publication of this case report and any accompanying images. A copy of the written consent is available for review by the Editor-in-Chief of this journal.

## Competing interests

The authors declare that they have no competing interests.

## Authors' contributions

CA, PS, and VK operated on the patient. VK, PS, and CA wrote the manuscript. All authors read and approved the final manuscript.
